# Reduced Relative Sensitivity of the Elecsys SARS-CoV-2 Antigen Assay in Saliva Compared to Nasopharyngeal Swabs

**DOI:** 10.3390/microorganisms9081700

**Published:** 2021-08-10

**Authors:** Annette Audigé, Jürg Böni, Peter W. Schreiber, Thomas Scheier, Roberto Buonomano, Alain Rudiger, Dominique L. Braun, Gerhard Eich, Dagmar I. Keller, Barbara Hasse, Christoph Berger, Huldrych F. Günthard, Amapola Manrique, Alexandra Trkola, Michael Huber

**Affiliations:** 1 Institute of Medical Virology, University of Zurich, 8057 Zurich, Switzerland; audige.annette@virology.uzh.ch (A.A.); boeni.juerg@virology.uzh.ch (J.B.); huldrych.guenthard@usz.ch (H.F.G.); manrique.amapola@virology.uzh.ch (A.M.); 2 Division of Infectious Diseases and Hospital Epidemiology, University Hospital Zurich, University of Zurich, 8091 Zurich, Switzerland; peterwerner.schreiber@usz.ch (P.W.S.); thomas.scheier@usz.ch (T.S.); Dominique.Braun@usz.ch (D.L.B.); barbara.hasse@usz.ch (B.H.); 3 Division of Infectious Diseases and Hospital Hygiene, Spital Limmattal, 8952 Schlieren, Switzerland; Roberto.Buonomano@spital-limmattal.ch; 4 Division of Medicine, Spital Limmattal, 8952 Schlieren, Switzerland; alain.rudiger@spital-limmattal.ch; 5 Division of Infectious Diseases, Hospital Hygiene and Occupational Medicine, Stadtspital Triemli, 8063 Zurich, Switzerland; Gerhard.Eich@triemli.zuerich.ch; 6 Emergency Department, University Hospital Zurich, 8091 Zurich, Switzerland; Dagmar.Keller@usz.ch; 7 Division of Infectious Diseases and Hospital Epidemiology, University Children’s Hospital Zurich, 8032 Zurich, Switzerland; christoph.berger@kispi.uzh.ch

**Keywords:** SARS-CoV-2, saliva, RT-PCR, antigen assay

## Abstract

Early identification and isolation of SARS-CoV-2-infected individuals is central to contain the COVID-19 pandemic. Nasopharyngeal swabs (NPS) serve as a specimen for detection by RT-PCR and rapid antigen screening tests. Saliva has been confirmed as a reliable alternative specimen for RT-PCR and has been shown to be valuable for diagnosing children and in repetitive mass testing due to its non-invasive collection. Combining the advantages of saliva with those of antigen tests would be highly attractive to further increase test capacities. Here, we evaluated the performance of the Elecsys SARS-CoV-2 Antigen assay (Roche) in RT-PCR-positive paired NPS and saliva samples (N = 87) and unpaired NPS (N = 100) with confirmed SARS-CoV-2 infection (Roche cobas SARS-CoV-2 IVD test). We observed a high positive percent agreement (PPA) of the antigen assay with RT-PCR in NPS, reaching 87.2% across the entire cohort, whereas the overall PPA for saliva was insufficient (40.2%). At Ct values ≤ 28, PPA were 100% and 91.2% for NPS and saliva, respectively. At lower viral loads, the sensitivity loss of the antigen assay in saliva was striking. At Ct values ≤ 35, the PPA for NPS remained satisfactory (91.5%), whereas the PPA for saliva dropped to 46.6%. In conclusion, saliva cannot be recommended as a reliable alternative to NPS for testing with the Elecsys Anti-SARS-CoV-2 Antigen assay. As saliva is successfully used broadly in combination with RT-PCR testing, it is critical to create awareness that suitability for RT-PCR cannot be translated to implementation in antigen assays without thorough evaluation of each individual test system.

## 1. Introduction

Severe Acute Respiratory Syndrome Coronavirus-2 (SARS-CoV-2) infection is routinely diagnosed by reverse-transcription polymerase chain reaction (RT-PCR) in nasopharyngeal swabs (NPS). In early infection, NPS are considered the optimal material for detection [1]. However, NPS collection requires trained personnel, exposes the personnel to a potential risk of nosocomial transmission, and is invasive. Saliva circumvents these problems and has proven advantageous for children, for whom conducting an NPS is challenging, as well as for adults, when frequent testing is required. A considerable advantage of saliva is that it does not require trained personnel for sampling and is amenable for home-collection. The overall sensitivity and thus suitability of saliva as an alternative to NPS has been differentially debated, with most studies reporting an acceptable performance of saliva despite a generally lower sensitivity and viral load compared to NPS [2,3,4,5,6,7,8,9,10,11,12,13,14,15,16], some studies showing a substantially lower sensitivity [17,18], and others reporting higher viral loads in saliva relative to NPS [19,20]. Differential saliva collection strategies, RT-PCR test systems, and small and heterogeneous study populations may cause discrepancies in the sensitivity of detecting SARS-CoV-2 infection in saliva. Therefore, controlled evaluations in large, well-defined cohorts are critical. To this end, we recently conducted a large-scale head-to-head comparison of SARS-CoV-2 detection by RT-PCR in NPS and saliva of adults and children in a test center setting [21]. We demonstrated that saliva is a reliable alternative specimen for the detection of SARS-CoV-2 by RT-PCR with particular advantages for testing children.

While saliva tested with RT-PCR has become a widely used strategy, additional testing options are in demand. Mass testing strategies would benefit from test formats that are rapid, simple, accurate, and non-invasive, which could be offered, for instance, by testing saliva with a SARS-CoV-2 antigen assay. Provided that saliva allows SARS-CoV-2 detection by antigen tests with sufficient sensitivity, this would combine the advantages of antigen testing, namely the ability to provide cost-effective results within minutes, with the advantages of non-invasive, self-collected saliva.

Here, we investigated the suitability of saliva as an alternative specimen for SARS-CoV-2 antigen detection by the Elecsys SARS-CoV-2 Antigen assay (Roche, Basel, Switzerland). We evaluated the performance of the assay for saliva samples in comparison to NPS by determining the agreement and correlation between the Elecsys Anti-SARS-CoV-2 Antigen assay and the cobas SARS-CoV-2 IVD test (Roche, Basel, Switzerland) stratified by sample type and SARS-CoV-2 E-gene Ct values. We confirmed that NPS samples have a high Positive Percent Agreement (PPA) between the antigen assay and the RT-PCR test. In contrast, the antigen assay had a much lower performance with saliva samples. In the latter sample type, SARS-CoV-2 could only be detected reliably in samples with Ct values ≤ 26. Saliva is thus not a suitable specimen in combination with the Roche Elecsys Anti-SARS-CoV-2 Antigen assay.

## 2. Materials and Methods

### 2.1. Study Population

Probed specimen were leftover from routine diagnostics and a previous study [21] approved with an ethics waiver by the Zurich Cantonal Ethics Commission (Req-2020-00398). No additional personal data beyond the usual information on symptoms and duration required by the Swiss Federal Office of Public Health for all SARS-CoV-2 tests in Switzerland were collected.

For nasopharyngeal sampling, conventional swab and virus transport medium (VTM)/universal transport medium (UTM) were used. Transport media included in-house VTM (HEPES, DMEM, FCS, antibiotics, antimycotics) and Virus Preservative Medium (Improviral) for paired PCR-positive NPS; Liquid amies preservation medium (Copan) for unpaired PCR-positive NPS; in-house VTM for paired PCR-negative NPS. Saliva collection was conducted as previously described [21]. Briefly, individuals were asked to clear the throat thoroughly and collect saliva up to three times into an empty saliva collection tube to collect 0.5–1 mL (approx. a teaspoon full). Immediately after saliva collection, VTM was added to the crude saliva and the content mixed through gentle tilting. Saliva was collected directly after NPS for paired analysis and both specimens were immediately sent for SARS-CoV-2 RT-PCR testing. Residual material was stored at −80 °C until use in the present study.

### 2.2. Specimen

RT-PCR tested (Roche cobas SARS-CoV-2 IVD test), cryo-preserved (−80 °C) NPS and saliva specimens were included. Paired SARS-CoV-2 NPS and saliva included positive (N = 87) and negative tested samples (N = 220) collected in the framework of a prior study that conducted a prospective head-to-head comparison of the specimen by RT-PCR [21]. In addition, SARS-CoV-2 positive NPS samples from routine diagnostic testing were included (N = 100).

### 2.3. Quantitative SARS-CoV-2 PCR

NPS and saliva were processed identically using the procedures established for NPS in the diagnostics laboratory of the Institute of Medical Virology, University of Zurich. A total of 500 µL of NPS or saliva in VTM were diluted in 500 µL of Nuclisens easyMAG Lysis Buffer (bioMérieux, Marcy-l’Étoile, France), centrifuged (2000 rpm, 5 min), and analyzed with the cobas SARS-CoV-2 IVD test (Roche, Basel, Switzerland) on a Cobas 6800. RT-PCR testing for paired NPS and saliva was done in parallel on the same day as part of a previously published study [21]. SARS-CoV-2 detection was further quantified using SARS-CoV-2 Frankfurt 1 RNA as a calibrator (European Virus Archive, 004N-02005) allowing to report both Ct and genome equivalents. E-gene Ct values were used for comparison. In one NPS sample, the E-gene Ct value was considerably higher than the ORF1-gene Ct value (37.31 vs. 26.8); in this case, the ORF1-gene Ct value was used for the analysis.

### 2.4. SARS-CoV-2 Antigen Assay

Samples were thawed at room temperature and analyzed with the Elecsys SARS-CoV-2 Antigen assay (Roche, Basel, Switzerland) on a cobas e 411 analyzer (Roche, Basel, Switzerland) according to the manufacturer’s instructions. Briefly, Elecsys SARS-CoV-2 Antigen is an electrochemiluminescence immunoassay, which detects the nucleocapsid (N) antigen of SARS-CoV-2. The assay uses an antibody sandwich principle. Results are recorded as the Cutoff Index (COI).

Saliva samples were treated identically to NPS. Single measurements were conducted.

### 2.5. Data Analysis

Positive Percent Agreement (PPA), Negative Percent Agreement (NPA), and Overall Percent Agreement (OPA) between the Elecsys Anti-SARS-CoV-2 Antigen assay and the cobas SARS-CoV-2 IVD test were calculated. PPA was determined and stratified by Ct values (PPAoverall and PPA < Ct). PPA, NPA, and OPA were calculated using R (version 4.0.2); 95% confidence intervals (CI, two-sided) were calculated with the epiR package (version 1.0.15). Analysis of correlation (Spearman correlation) between the E-gene Ct value and antigen assay Cutoff Index (COI) and linear regression analysis were performed with GraphPad Prism (version 8.4.3). *p* values < 0.05 were considered statistically significant. All raw data are available in Supplemental Table S1.

## 3. Results

### 3.1. Lower Qualitative Agreement of the Elecsys Anti-SARS-CoV-2 Antigen Assay with RT-PCR in Saliva Than in NPS

The Elecsys SARS-CoV-2 Antigen assay has been established for the detection of SARS-CoV-2 in nasopharyngeal and oropharyngeal swab samples from patients with signs and symptoms suggestive of COVID-19 or known or suspected exposure to SARS-CoV-2 (Elecsys SARS-CoV-2 Antigen Assay Method sheet v1 (December 2020)). Here, we probed its capacity in detecting SARS-CoV-2 in saliva by comparing the performance in specimens previously tested by RT-PCR (cobas SARS-CoV-2 IVD). PCR-positive samples included paired NPS-saliva samples (N = 87) and unpaired NPS samples (N = 100). PCR-negative samples comprised paired NPS-saliva samples (N = 220). Symptomatic and asymptomatic donors were included (Table 1).

We first established the positive percent agreement (PPA) of the Elecsys Anti-SARS-CoV-2 Antigen assay using NPS or saliva with the cobas SARS-CoV-2 IVD test. The overall PPA for NPS (including paired and unpaired NPS; N = 187) was 87.2%, with 100% PPA for samples with Ct values ≤ 28 and respectable 91.5% PPA for samples with Ct values ≤ 35 (Table 2). In the paired NPS-saliva cohort (N = 87), these values were similar for NPS, reaching PPAoverall = 86.2%, PPA<Ct28 = 100%, and PPA<Ct35 = 93.7%, whereas saliva (N = 87) performed clearly less well, yielding PPAoverall = 40.2%, PPA<Ct26 = 100%, PPA<Ct28 = 91.2%, and PPA<Ct35 = 46.6%, only. Thus, a reliable detection of SARS-CoV-2 in saliva with the Roche Elecsys Anti-SARS-CoV-2 Antigen assay was only possible for samples with Ct values ≤ 26, showing the lower performance of the antigen assay with saliva compared to NPS.

To assess the specificity of the Roche Elecsys Anti-SARS-CoV-2 Antigen assay, we next determined the Negative Percent Agreement (NPA) and Overall Percent Agreement (OPA) of the Roche Elecsys Anti-SARS-CoV-2 Antigen assay in reference to the cobas SARS-CoV-2 IVD test. NPA was 100% and 99.5% for NPS and saliva, respectively (Table 3). The lower NPA in saliva was due to a single saliva sample that yielded a positive result (COI = 1.41). A repeat analysis of this sample retrieved a negative result (COI = 0.612). As all other samples were measured only once, the first positive result was considered for analysis. In sum, the Elecsys Anti-SARS-CoV-2 Antigen assay showed a high specificity both in NPS and saliva. The Overall Percent Agreement (OPA) was 94.1% and 82.7% for NPS and saliva, respectively (Table 3).

### 3.2. Low Qualitative Agreement of Antigen Measurements between Paired NPS and Saliva

To assess the capacity of the antigen assay to detect SARS-CoV-2 more directly, we next determined the agreement between the antigen assay results obtained for NPS and saliva in the paired sample cohort. This analysis yielded very low percentages for PPA (41.3%), NPA (66.7%), and OPA (44.8%), underlining the limited performance of saliva testing with the antigen assay (Table 4).

### 3.3. Lower Quantitative Agreement of the Elecsys Anti-SARS-CoV-2 Antigen Assay with RT-PCR Diagnosis in Saliva Than in NPS

**Figure 1:**
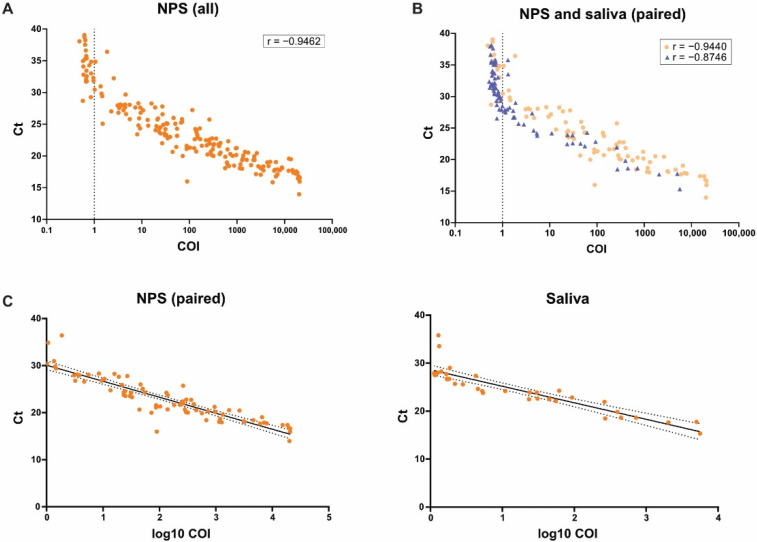
Analysis of correlation between Roche Elecsys Anti-SARS-CoV-2 Antigen assay and cobas SARS-CoV-2 IVD test for NPS and saliva samples and linear regression. Spearman correlation between Elecsys Anti-SARS-CoV-2 Antigen assay COI values (*X* axis, log scale, cutoff indicated by dotted line) and cobas SARS-CoV-2 IVD test Ct values (*Y* axis) was analyzed for all NPS (N = 187 *, *p* < 0.0001) (**A**) and paired NPS (orange circles, N = 87 *, *p* < 0.0001) and saliva samples (blue triangles, N = 87, *p* < 0.0001) (**B**). * Including one sample for which the ORF1-gene Ct value was used. Linear regression analysis of only positive antigen tests (COI values ≥ 1) was performed for NPS (left plot) and saliva (right plot); black solid line shows the regression line; dotted black lines show the 95% confidence bands (**C**).

We next evaluated the quantitative agreement of the antigen assay and RT-PCR for both NPS and saliva specimens. Antigen measurements (COI values) and RT-PCR (E-gene Ct values) correlated generally well in both specimen types (Figure 1A); however, saliva yielded a lower correlation coefficient compared to NPS (Figure 1B).

Linear regression of only positive antigen tests (COI values ≥ 1) yielded different E-gene Ct cutoffs in NPS vs. saliva (Figure 1C). This shift of 1.45 Ct could potentially indicate a matrix effect of saliva that affects detection in the antigen assay at equal sensitivity as in NPS.

We previously reported that Ct values were on average 4.87 higher in saliva than in the corresponding NPS, which corresponds to, on average, 30-fold lower viral loads in saliva [21]. This was also evident in the paired NPS-saliva cohort analyzed in the present study, where we observed, on average, 4.6 higher Ct values in saliva (data not shown).

### 3.4. Low Capacity of Saliva Antigen Testing in Clinical Diagnosis

The current gold standard for SARS-CoV-2 diagnosis remains RT-PCR detection in NPS, and the sensitivity of other test formats, including antigen tests, are evaluated in reference to it. For SARS-CoV-2 antigen detecting rapid diagnostic tests (Ag-RDTs) of respiratory specimens, the WHO defines as the minimum performance requirements a sensitivity of ≥80% and a specificity of ≥97% compared to a nucleic acid amplification test (NAAT) reference assay (https://www.who.int/publications/i/item/antigen-detection-in-the-diagnosis-of-sars-cov-2infection-using-rapid-immunoassays, accessed on 12 May 2021). Considering that saliva contains less virus than NPS, the performance of saliva antigen testing needs to be put into relation to corresponding levels of the virus in NPS to yield relevant information.

**Figure 2:**
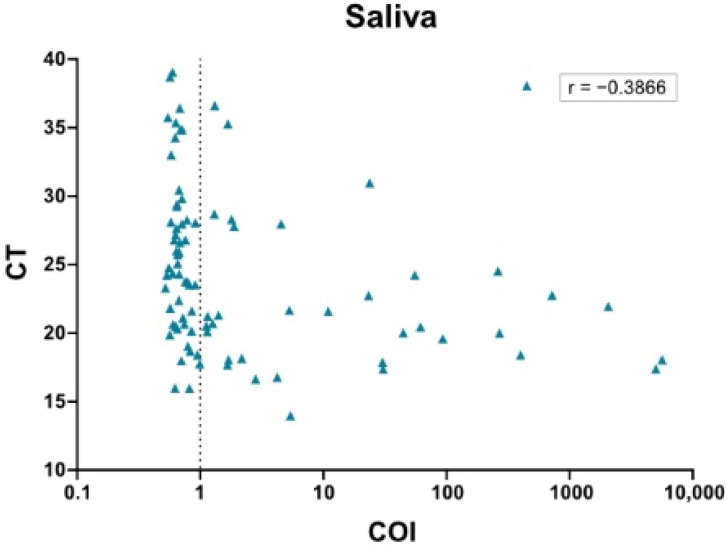
Analysis of correlation between Roche Elecsys Anti-SARS-CoV-2 Antigen assay and cobas SARS-CoV-2 IVD test for saliva samples based on Ct values of corresponding NPS. Spearman correlation between Elecsys Anti-SARS-CoV-2 Antigen assay COI values (*X* axis, log scale, cutoff indicated by dotted line) and cobas SARS-CoV-2 IVD test Ct values of the corresponding NPS (*Y* axis) was analyzed for saliva samples (N = 87, *p* = 0.0002).

We therefore assessed the diagnostic agreement between NPS RT-PCR as a reference and the paired saliva antigen assay results. The analysis highlighted the markedly reduced clinical performance of saliva antigen testing. PPA overall was insufficient (40.2%) (Table 5). Even for samples with Ct values (of the corresponding NPS) ≤ 26, PPA was only 47.4%. In line with these data, analysis of correlation between COI values of saliva and Ct values of the corresponding NPS yielded a very low correlation coefficient (Figure 2). Thus, saliva testing with the Elecsys Anti-SARS-CoV-2 Antigen assay has overall a low capacity in clinical diagnosis.

**Figure 3:**
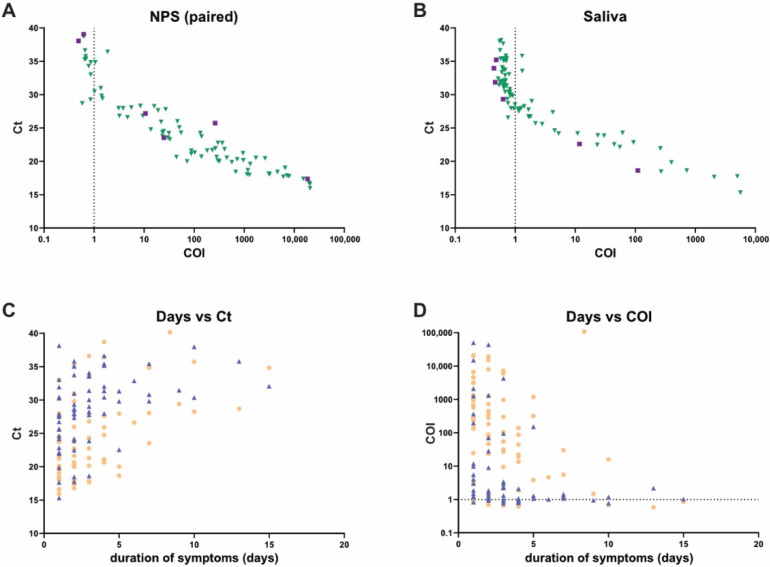
SARS-CoV-2 detection in asymptomatic and symptomatic disease stages. (**A**,**B**) Analysis of paired NPS (**A**) and saliva (**B**) samples from asymptomatic (N = 6) and symptomatic (N = 77) individuals that tested positive for SARS-CoV-2 by RT-PCR. Roche Elecsys Anti-SARS-CoV-2 Antigen assay results (COI value) and corresponding cobas SARS-CoV-2 IVD test results (Ct value) are depicted. Cutoff of the antigen test is indicated by a dotted line. Green triangles: asymptomatic, violet squares: symptomatic. Comparison of duration of symptoms (days) and outcome of cobas SARS-CoV-2 IVD test (**C**) or Roche Elecsys Anti-SARS-CoV-2 Antigen assay (**D**) in paired symptomatic SARS-CoV-2 positive NPS (orange circles, N = 65) and saliva (blue triangles, N = 65) samples. For 12 of the 77 symptomatic patients, no information on duration of symptoms was available.

The study was designed to assess the analytical performance of saliva antigen testing. While an assessment of the performance of the antigen test in saliva at different disease stages would require a dedicated study protocol and enrollment, we had basic data on symptom status available for our cohort (Table 1) that allowed a first analysis (Figure 3). Comparing diagnostic performance of RT-PCR and the antigen test in paired NPS and saliva samples that tested positive in symptomatics (N = 77) and asymptomatics (N = 6), we observed the expected lower sensitivity of the antigen test in samples with lower viral load (higher Ct value) irrespective of disease pattern and duration (Figure 3).

## 4. Discussion

In the present study, we evaluated the suitability of saliva for detection of SARS-CoV-2 with the Elecsys Anti-SARS-CoV-2 Antigen assay. The combination of a specimen that can be easily and safely obtained with an accurate and rapid detection method would expand the range of strategies for diagnosing early SARS-CoV-2 infection. NPS is considered the optimal material for detection of SARS-CoV-2 in early infection [1]. However, its collection requires trained personnel, which are exposed to a potential risk of nosocomial transmission and may create discomfort for the patient. Saliva circumvents these problems, but its sensitivity and thus suitability has been differentially reported [2,3,4,5,6,7,8,9,10,11,12,13,14,15,16,17,18,19,20,22,23,24,25,26,27,28,29,30,31,32,33,34,35,36,37,38,39,40,41]. We recently conducted a large-scale head-to-head comparison of SARS-CoV-2 detection by RT-PCR in NPS and saliva and could show that saliva is a valid alternate specimen for SARS-CoV-2 detection by RT-PCR with particular advantages for testing children [21].

The majority of the currently used antigen tests are Ag-RDTs of respiratory specimens to be used at the Point of Care (POC). A recent Cochrane study reported a sensitivity of only 56.2% (95% confidence interval (CI): 29.5–79.8%) for point-of-care antigen tests [33]. Several studies have evaluated saliva as an alternative to NPS for detection of SARS-CoV-2 with antigen tests, including automated tests [11,12,13,14,15,20,36,37,38,40,42,43,44]. However, none of the current Emergency Use Authorization (EUA) tests for SARS-CoV-2 antigen detection is authorized for saliva.

The Elecsys Anti-SARS-CoV-2 Antigen assay allows for a rapid detection of the SARS-CoV-2 nucleocapsid protein in NPS and oropharyngeal swab samples with a good sensitivity for symptomatic patients. Our study confirms the high agreement between the Elecsys Anti-SARS-CoV-2 Antigen assay and the cobas SARS-CoV-2 IVD test for NPS samples as reported by the manufacturer. Notably, in our study cohort, the PPA (86.2% for all NPS samples, N = 187) was even higher than the relative sensitivity reported by the manufacturer (PPA of 60.5%, N = 390). This was also evident from the observed thresholds of detection, which were for a PPA of 100% at Ct ≤ 28 in our cohort compared to Ct < 26 reported by the manufacturer.

In contrast to the NPS samples, the Elecsys Anti-SARS-CoV-2 Antigen assay performed much less well with saliva. For the saliva samples, the PPA (with RT-PCR in saliva) was overall only 40.2%, and SARS-CoV-2 could be detected reliably only in samples with Ct values ≤ 26. It is important to note that saliva collection was not tailored for the use with this specific assay but was based on a method that we optimized for detection of SARS-CoV-2 by RT-PCR and entails a dilution of saliva upon collection. The ability to use the same material for different test systems is preferable in diagnostic laboratories, because it provides flexibility to choose different test systems for reanalysis.

Our study shows that lower sensitivity can occur in antigen tests using saliva as a medium and highlights the need for developers to establish appropriate protocols for saliva testing. It must be considered that a reduced sensitivity of SARS-CoV-2 antigen testing in saliva is likely not restricted to the assay evaluated here, as several studies noted lower sensitivity in saliva [11,12,13,14,15,20,22,36,37,38,39,40,41,42,43,44,45]. Alternate saliva sampling strategies that allow concentrated saliva processing may thus be of advantage in combination with antigen tests to overcome the sensitivity restrictions. Nevertheless, processing undiluted saliva is challenging due to its viscosity and may not be applicable for automated processing. Optimization of saliva pretreatment will be most effective if tailored by the manufacturers to the respective assay system to avoid interference with detection reagents.

Our results show a clear advantage of NPS as a specimen in combination with the probed antigen test. This is in concordance with other antigen test systems which, in general, have shown lower sensitivity compared to NPS [35,42,43,44,45,46,47,48,49,50,51,52,53,54,55,56]. One of the automated antigen tests previously evaluated for saliva samples is the Lumipulse G SARS-CoV-2 Ag assay, a chemiluminescent enzyme immunoassay which received the CE marking for qualitative and quantitative detection of the SARS-CoV-2 N antigen on both saliva and NPS samples. Sensitivities relative to the corresponding molecular reference test ranged from 41.3% to 88.9%; specificities were reported in four of the five studies and ranged from 96.9% to 98.6% [42,43,44,46,51]. It has to be noted, however, that in the study reporting the highest sensitivity (i.e., 88.9%), only nine RT-PCR-positive saliva samples were evaluated. Another automated antigen test evaluated for saliva is the Simoa SARS-CoV-2 N Protein Antigen Test, which is an EUA for the qualitative detection of the N antigen from SARS-CoV-2 in NPS specimens. PPA and NPA of this assay with molecular testing were 92.3% and 98.1% (N = 26) for days 1–7 [53]. Thus, compared to these two SARS-CoV-2 antigen assays, the Elecsys Anti-SARS-CoV-2 Antigen assay has a lower sensitivity but higher specificity. The sensitivity comparison must, however, be treated with caution, as sensitivity will vary depending on the virus load (Ct) of the included test samples.

The current study was designed as proof of principle analysis to define the capacity of the Elecsys antigen tests in detecting SARS-CoV-2 in saliva. We based our analysis on comparing efficacy of the test in NPS and saliva with a known viral load. We ascertained a representative range of viral loads in the test cohort samples. The study was not designed to assess the performance of the antigen test in saliva of specific patient groups (children versus adults) or disease stages (asymptomatic versus symptomatic). While extended analyses on the use of saliva antigen testing in different disease stages would have been of interest, the low sensitivity we observed in saliva rendered these study extensions unfortunately unnecessary.

The lower sensitivity in saliva with the antigen test probed here highlights the general importance of carefully evaluating saliva as a test material. As we showed, conducting head-to-head comparisons of NPS and saliva for both PCR and antigen test is critical to dissect if matrix effects in saliva exist. As only few studies have assessed saliva in the context of SARS-CoV-2 antigen tests, and those that have, recorded variable results [35,41,42,43,44,45,46,47,48,49,50,51,52,53,54,55,56], it can currently not be excluded that antigen tests generally show a reduced sensitivity across diverse tests systems. Alternatively, these effects may be test-dependent and can be modest enough to allow application in mass testing as a recent study suggests [41].

In sum, there is a general agreement based on published studies and the data presented here that SARS-CoV-2 antigen tests in saliva are less sensitive and, thus, should only be considered when a clear benefit for clinical diagnosis can be established.

## Figures and Tables

**Table 1:** Donor demographics.

SARS-CoV-2	Positive	Positive	Negative
	Unpaired NPS (N = 100)	Paired NPS/Saliva (N = 87)	Paired NPS/Saliva (N = 220)
Male/Female (%)	45 (45%)/ 55 (55%)	56 (64%)/ 31 (36%)	117 (53%)/ 103 (47%)
Age median (range)	52 (13–98)	35 (16–76)	29 (16–77)
Symptomatic (%)	94 (94%)	77 (88%)	167 (76%)
Asymptomatic (%)	5 (5%)	6 (7%)	50 (23%)
No information on symptoms (%)	1 (1%)	4 (5%)	3 (1%)
Median days of symptoms (range)	2 (1–28)	2 (1–15)	3 (1–14)
No information on duration of symptoms (%)	2 (2%)	16 (21%)	33 (20%)

**Table 2:** Overview of positive percent agreement (PPA) of the Roche Elecsys Anti-SARS-CoV-2 Antigen assay with the Roche cobas SARS-CoV-2 IVD test in NPS and saliva.

Cobas E-Gene Ct Value ^a^	NPS (All)		NPS (Paired)		Saliva	
	N (cum. ^b^)	PPA, % (95% CI)	N (cum.)	PPA, % (95% CI)	N (cum.)	PPA, % (95% CI)
≤26	134	100 97.3–100	57	100 93.7–100.0	22	100 84.6–100
≤27	138	100 97.4–100	60	100 94.0–100.0	26	96.2 80.4–99.9
≤28	150	100 97.6–100	65	100 94.5–100.0	34	91.2 76.3–98.1
≤29	156	99.4 96.5–100	70	98.6 92.3–100	37	86.5 71.2–95.5
≤30	160	98.8 95.6–99.8	73	97.3 90.4–99.7	42	78.6 63.2–89.7
≤31	162	98.8 95.6–99.9	75	97.3 90.7–99.7	50	66.0 51.2–78.8
≤32	165	97.0 93.1–99	75	97.3 90.7–99.7	60	55.0 41.6–67.9
≤33	170	94.7 90.2–97.6	75	97.3 90.7–99.7	65	50.8 38.1–63.4
≤34	172	93.6 88.8–96.8	76	96.1 88.9–99.2	72	47.2 35.3–59.3
≤35	177	91.5 86.4–95.2	79	93.7 85.8–97.9	73	46.6 34.8–58.6
Overall (≤40)	187	87.2 81.5–91.6	87	86.2 77.1–92.7	87	40.2 29.9–51.3

^a^ sample type-specific Ct values; ^b^ cumulative.

**Table 3:** NPA and OPA of the Roche Elecsys Anti-SARS-CoV-2 Antigen assay with the Roche cobas SARS-CoV-2 IVD test.

	N	NPA, % (95% CI)	OPA, % (95% CI)
NPS	220 (NPA) 407 (OPA)	100 98.3–100	94.1 91.4–96.2
Saliva	220 (NPA) 307 (OPA)	99.5 97.4–100	82.7 78.0–86.8

**Table 4:** PPA, NPA, and OPA between Roche Elecsys Anti-SARS-CoV-2 Antigen results in paired NPS and saliva.

N NPS/Saliva	PPA, % (95% CI)	NPA, % (95% CI)	OPA, % (95% CI)
87/87	41.3 30.1–53.3	66.7 34.9–90.1	44.8 34.1–55.9

**Table 5:** PPA of the Roche Elecsys Anti-SARS-CoV-2 Antigen assay with the Roche cobas SARS-CoV-2 IVD test in saliva based on Ct values of corresponding NPS.

Cobas E-Gene Ct Value ^a^	Saliva	
	N (cum.)	PPA, % (95% CI)
≤26	57	47.4 34.0–61.0
≤27	60	45.0 32.1–58.4
≤28	65	44.6 32.2–57.5
≤29	70	44.3 32.4–56. 7
≤30	73	42.5 31.0–54.6
≤31	75	42.7 31.3–54.6
≤32	75	42.7 31.3–54.6
≤33	75	42.7 31.3–54.6
≤34	76	42.1 30.9–54.0
≤35	79	40.5 29.6–52.1
Overall (≤40)	87	40.2 29.9–51.3

^a^ Ct values of corresponding NPS.

## Data Availability

All raw data are provided as Supplementary Materials.

## Supplementary Materials

The following are available online at https://www.mdpi.com/article/10.3390/microorganisms9081700/s1, Table S1: Raw data and donor demographics.
